# Consommation des produits lactés chez l’enfant et l’adolescent marocain de 2 à 16 ans: une étude monocentrique Consumption of milk products among Moroccan children and adolescents aged 2-16 years: a monocentric study

**DOI:** 10.11604/pamj.2017.28.125.9533

**Published:** 2017-10-10

**Authors:** Hajar Berrani, Asmae Mdaghri Alaoui, Said Ettair, Nezha Mouane, Amal Thimou Izgua

**Affiliations:** 1 Equipe de Recherche en Nutrition et Sciences de l’Alimentation, Hôpital d’enfants, Rabat, Maroc; 2 Service de Pédiatrie III, Faculté de Médecine et Pharmacie, Université Mohammed V, Rabat, Maroc; 3 Service de Néonatologie, Pédiatrie V, Faculté de Médecine et Pharmacie, Université Mohammed V, Rabat, Maroc

**Keywords:** Lait, produits laitiers, facteurs associés, Milk, milk products, associated factors

## Abstract

**Introduction:**

Evaluer la consommation quotidienne des produits laitiers dans une population d’enfants marocains et déterminer les facteurs associés pouvant influencer cette consommation.

**Méthodes:**

Etude prospective du 1^er^ octobre 2013 au 31 avril 2014. Les enfants âgés entre 2 et 16 ans ont été inclus. Le recrutement a eu lieu dans la ville de Fès. Le recueil des données s’est fait à l’aide d’un questionnaire fréquentiel. Les parents et les enfants inclus ont été interrogés sur la consommation des produits laitiers et les facteurs socio-démographiques avec une évaluation anthropométrique des enfants. L’association des variables à la consommation des produits laitiers a été analysée en analyse univariée et multivariée par un modèle de régression logistique.

**Résultats:**

L’enquête alimentaire avait intéressé 286 enfants dont 151 filles (52,8 %) et 131 garçons (45,8%). Les enfants âgés de 2 à 3 ans représentaient 26,4 %, ceux âgés de 4 à 7 ans 28,9 %, ceux âgés de 7 à 9 ans 18,3 % et les adolescents âgés de 10 à 16 ans 26,4 %. Les enfants consommaient en moyenne 2.5±1 produits laitiers par jour. Les enfants consommaient au moins 3 produits laitiers par jour dans 57,8% chez les enfants âgés de 2 à 3 ans, 53,6% chez les enfants âgés de 4 à 6 ans, 40% chez les enfants âgés de 7 à 9 ans et 41.2% chez les enfants âgés de 10 à 16 ans. Les facteurs associés à la consommation de trois produits laitiers minimum par jour en analyse univariée étaient le niveau d’instruction maternel analphabète p < 0.001 OR = 0.1 et primaire p = 0.002 OR = 0.1, le niveau socioéconomique familial moyen p < 0.001 OR = 3, l’âge p= 0.01 OR = 0.9 et l’indice de masse corporelle normal p = 0.01 OR = 2.5 et > 90° percentiles p < 0.001 OR = 6. Il existe un lien positif entre l’indice de masse corporelle > 90° percentiles p = 0.01 OR = 3.9 est et la quantité consommée des produits laitiers et négatif avec le faible niveau de scolarité maternel analphabète p = 0.008 OR = 0.1 et primaire p = 0.009 OR = 0.1.

**Conclusion:**

La consommation du lait et des autres produits laitiers était inappropriée en particulier chez l’enfant âgé de 7 à 9 ans et l’adolescent de 10 à 16 ans. Le faible niveau d’éducation maternel et un indice de masse corporelle supérieur au 90° percentiles était des facteurs indépendamment associés à la consommation de moins de 3 produits laitiers par jour. La sensibilisation des parents et des enfants sur l’intérêt du lait et de ses dérivés dans l’alimentation de l’enfant est indispensable.

## Abstract (en)

**Introduction:**

This study aimed to evaluate the daily consumption of milk products in a population of Moroccan children and to determine the factors influencing this consumption.

**Methods:**

We conducted a prospective study from 1 October 2013 to 31 April 2014. Children aged between 2 and 16 years were included in the study. The participants were recruited in the city of Fez. Data were collected using a frequency questionnaire. Enrolled parents and children were interviewed on their consumption of milk products and on sociodemographic factors. Children underwent anthropometric examination. The association between variables in milk products consumption was analyzed using univariate and multivariate analysis with logistic regression model.

**Results:**

Food survey involved 286 children: 151 girls (52.8%) and 131 boys (45.8%). Children aged 2 to 3 years accounted for 26.4%, those aged 4 to 7 years accounted for 28.9%, those aged 7 to 9 years accounted for 18.3% and adolescents aged 10 to 16 years accounted for 26.4%. Children consumed on average 2.5±1 milk products per day. 57.8% of children aged 2 to 3 years, 53.6% of children aged 4 to 6 years, 40% of children aged 7 to 9 years and 41.2% of children aged 10 to 16 years consumed at least 3 milk products per day. The factors associated with the consumption of at least three milk products per day in univariate analysis were an illiterate maternal education level p < 0.001 OR= 0.1 and an elementary maternal education level p = 0.002 OR = 0.1, a medium familial socio-economic status p < 0.001 OR = 3, age p = 0.01 OR = 0.9 and a normal body mass index p = 0.01 OR = 2.5 and > 90° percentiles p < 0.001 OR= 6. There was a positive correlation between a body mass index > 90° percentiles p= 0.01 OR = 3.9 and the quantity of consumed milk products while there was a negative correlation between a body mass index > 90° percentiles p = 0.01 OR = 3.9 and a low maternal schooling: illiterate p = 0.008 OR= 0.1 elementary p = 0.009 OR = 0.1.

**Conclusion:**

The consumption of milk and of other milk products was inappropriate in particular among children aged 7 to 9 years and adolescents aged 10 to 16 years. Low maternal schooling and a body mass index higher than 90° percentiles were factors independently associated with the consumption of less than 3 milk products per day. The awareness of parents and children about the role of the milk and its derivatives in children diet is essential.

## Introduction

Le lait et les produits laitiers (yaourts, fromage, lait fermentés, dessert lactés, …) sont une source d’énergie, de protéines, de matières grasses, de calcium et des micronutriments. Ils constituent la principale et la meilleure source alimentaire de calcium, ils apportent plus que la moitié des besoins quotidiens du calcium [1, 2]. Les produits laitiers sont également une source des micronutriments comme le phosphore, le magnésium, l’iodine, le zinc, le potassium, la vitamine A, la vitamine D, la vitamine B12 et la riboflavine [3]. Les apports calciques journaliers conseillés en France chez l’enfant normal sont de l’ordre de 500 mg pour l’enfant de 1 à 3 ans et de 700 mg entre 4 et 6 ans. Cependant, ces apports sont de l’ordre de 900 mg entre 7 et 9 ans et de 1200 mg à partir de l’âge de 10 ans [4]. Une association positive existe entre le pic de la densité minérale osseuse à l´âge adulte et la consommation de produits laitiers pendant l’enfance [5]. Il est recommandé d’apporter 2 à 3 produits laitiers par jour chez l’enfant de moins de 9 ans et 3 à 4 chez l’enfant plus âgé [6]. Cependant, les enquêtes de consommation dans de nombreux pays révèlent une faible consommation du lait et de ses dérivés qui n’atteint pas les apports nutritionnels recommandés chez l’enfant avec une tendance à la diminution de cette consommation avec l’âge dans différents pays [2, 7, 8]. Au Maroc, aucune enquête nationale ne s’est intéressée à la consommation des produits laitiers chez l’enfant, d’ou l’intérêt de notre travail dont l’objectif principal est d’évaluer la consommation quotidienne des produits laitiers chez une population d’enfants marocains. L’objectif secondaire de notre étude est de déterminer les facteurs sociodémographiques et anthropométriques associés pouvant influencer cette consommation.

## Méthodes

Il s’est agi d’une étude transversale à visée descriptive et analytique d’une durée étalée du premier octobre 2013 au 31 avril 2014 et visant à évaluer la consommation quotidienne des produits laitiers chez les enfants de la ville de Fès. Les critères d’inclusion étaient l’âge de 2 à 16 ans et le lieu de résidence dans la ville de Fès. Les critères d’exclusion étaient une allergie aux protéines de lait de vache ou une autre allergie alimentaire, une intolérance au lactose, une pathologie chronique ou une prise médicamenteuse.

Le recrutement a eu lieu aux centres de santé dans la ville de Fès. En se basant sur la prévalence de la malnutrition chronique chez l’enfant marocain selon l’enquête nationale [9] sur la population et la santé publique en 2011 de 14,9 %, avec un intervalle de confiance de 95% et une marge d’erreur de 5%, la taille de l’échantillon qui doit être incluse dans cette étude est de 197. Dans notre travail, 286 enfants étaient recrutés. L’échantillonnage s’est effectué de façon non randomisée. Les enfants qui se sont présentés aux centres de santé pour une consultation pour une pathologie intercurrente, une consultation de médecine scolaire ou lors des campagnes de vaccination (le vaccin rougeole rubéole et le rappel du vaccin diphtérie-tétanos-coqueluche-poliomyélite à l’âge de 5 ans et le vaccin antitétanique pour les adolescentes de 15 ans) et qui répondaient aux critères d’inclusion étaient recensé du lundi au vendredi lors des heures ouvrables après l’obtention du consentement oral des parents. Le recueil des données s’est fait à l’aide d’un questionnaire fréquentiel rempli par un médecin. Les parents et les enfants inclus ont été interrogés sur la consommation de lait (lait pur, boissons lactées, etc.) et les produits laitiers (yaourt, petit suisse, fromage blanc, à pâte cuite, à pâte molle, etc.) dans chaque repas (petit déjeuner, collation, déjeuner, goûter, diner). L’enregistrement des produits laitiers s’est fait par une quantification en unités ménagères. Les portions des produits laitiers ont été définies comme suivants: un verre de lait (125 ml), un yaourt (110 g), un fromage frais (240 g) et la crème-dessert (250 ml).

L’analyse statistique a été réalisée en utilisant le logiciel SPSS version 13.0. Les variables qualitatives ont été exprimées en pourcentage avec leur effectif. Les variables quantitatives ont été exprimées en moyenne et écart-type si la distribution était gaussienne ou médiane avec quartile lorsqu’elle ne suivait pas la loi normale. La fréquence de la consommation des produits laitiers journaliers entre les différents groupes d’âge était étudiée par un test de Chi 2. La quantité consommée des produits laitiers et l’apport calcique journaliers entre les différents groupes d’âge était étudiée par un test d’analyse de variance ANOVA. L’association des variables à la consommation de 3 produits laitiers au moins par jour a été recherchée par une analyse univariée selon un modèle de régression logistique. Les facteurs significatifs (le degré de signification statistique a été retenu pour p < 0,05) étaient inclus dans l’analyse multivariée par le modèle de régression logistique. L’accord du comité d’éthique de la faculté de médecine de Rabat a été obtenu.

## Résultats

L’enquête alimentaire avait intéressé 286 enfants dont 151 filles (52,8 %) et 131 garçons (45,8%). Concernant le profil sociodémographique des parents ; l’âge moyen des mères des enfants était de 35,5±8,1 ans, les mères étaient analphabètes dans 53,2%, n’ayant aucune activité professionnelle dans 93,3 % et le niveau socio-économique familial était bas dans 70,1%. L’effectif des enfants âgés de 2 à 3 ans étaient 75 (26,4 %), ceux âgés de 4 à 7 ans 82 (28,9 %), ceux âgés de 7 à 9 ans 52 (18,3 %) et les adolescents âgés de 10 à 16 ans 75 (26,4 %). Les enfants consommaient du lait chaque jour dans 72,8 %. Les enfants consommaient le lait seul dans 76,6 %, aromatisés dans 16,8 % des cas et les deux dans 6,6 % des cas. Les enfants consommaient les autres produits laitiers tous les jours dans 88 % des cas. Les produits laitiers sont consommés principalement au moment du petit déjeuner chez 63,7% (Tableau 1). Les enfants avaient un indice de masse corporelle (IMC) inférieur aux 3 percentiles chez 23,3%, un IMC normal dans 63,5% et un IMC supérieur aux 90 percentiles dans 13,3%. Les enfants consommaient en moyenne 2.5±1 produits laitiers par jour, ils consommaient 4 produits laitiers par jour dans 22,3%, 3 produits laitiers par jour dans 26,7%, 2 produits laitiers dans 32,4% et un seul produit laitier par jour dans 18,6% des cas (Tableau 2). La consommation journalière moyenne du lait était de 302.6±183.2 ml pour les enfants âgés de 2 à 3 ans, de 242±120.2 ml pour les enfants âgés de 4 à 6 ans, de 172±65.4 ml pour les enfants âgés de 7 à 9 ans et de 176.1±91.6 ml pour les enfants âgés de 10 à 16 ans (Tableau 3). La consommation journalière moyenne du yaourt était de 168±89.5 g pour les enfants âgés de 2 à 3 ans, de 156±96.3 g pour les enfants âgés de 4 à 6 ans, 117.8±66.4 g pour les enfants âgés de 7 à 9 ans et de 129.3±55.1 g pour les enfants âgés de 10 à 16 ans. La consommation journalière des autres produits laitiers était de 166.7±34.1 g pour les enfants âgés de 2 à 3 ans, de 125 [62.5; 250] pour les enfants âgés de 4 à 6 ans, de 107 [0;125] g pour les enfants âgés de 7 à 9 ans et de 125±91.3 g pour les enfants âgés de 10 à 16 ans (Tableau 2). Les facteurs associés à la consommation de trois produits laitiers minimum par jour en analyse univariée: le niveau d’instruction maternel analphabète p < 0.001 OR = 0.1 et primaire p = 0.002 OR = 0.1, le niveau socioéconomique familial moyen p < 0.001 OR = 3, l’âge p = 0.01 OR=0.9 et l’indice de masse corporelle normal p= 0.01 OR= 2.5 et > 90° percentiles p <0.001 OR = 6 (Tableau 4). En analyse multivariée, le faible niveau de scolarité maternel: analphabète p = 0.008 OR = 0.1, primaire p = 0.009 OR= 0.1 et l’indice de masse corporelle > 90° percentiles p = 0.01 OR = 3.9 sont indépendamment des facteurs de risque à la consommation de trois produits laitiers minimum par jour (Tableau 5).

**Tableau 1:** Répartition des différents produits laitiers selon leurs moments de consummation

Repas	Lait n (%)	Yaourt n (%)	Fromage n (%)	Autres n (%)
Petit déjeuner	110 (38,5)	22 (7,7)	81 (28,3)	2 (1)
Collation	7 (2,4)	54 (18,9)	10 (3,5)	24 (8,4)
Déjeuner	1(0,3)	5 (1,7)	6 (2,1)	16 (5,5)
Gouter	17 (5,9)	89 (31,1)	70 (24,5)	44 (15,4)
Diner	4 (1,4)	11 (3,8)	4 (1,4)	20 (6,9)

**Tableau 2:** Fréquence de consommation des produits laitiers journalier en fonction des groupes d’âge et du sexe

		Groupe d'âge							
		2 à 3 ans		4 à 6 ans		7 à 9 ans		10 à 16 ans	
		F	M	F	M	F	M	F	M
Produits laitiers/ jour	1	3 (13)	3(13)	7(30,4)	7(30,4)	7(30,4)	7(30,4)	6(26,1)	6(26,1)
	2	9(23,7)	12(29,3)	10 (26,3)	8(19,5)	8 (21,1)	4(9,8)	11 (28,9)	17(41,5)
	3	7 (25)	8(21,6)	9 (32,1)	12(32,4)	3 (10,7)	7(18,9)	9 (32,1)	10(27)
	≥4	11 (44)	11(39,3)	7 (28)	8(26,8)	2 (18)	5(17,9)	5 (20)	4(14,3)

M : masculin, F : féminin p=0.041

**Tableau 3:** Quantité consommée des produits laitiers en fonction des groupes d’âge et du sexe

		Lait (ml)	Yaourt (g)	Fromage (g)	Autres (g)
2 à 3 ans	F	275 [250 ; 450]	110[110 ; 220]	395.3±188.6	125 [62 ; 312.5]
	M	250 [125 ; 356]	168.3±80.3	353.7±167.2	160.7±94.5
	Total^+^	302.6±183.2	168±89.5	373.3±176.3	125 [134 ; 166]
4 à 6 ans	F	236±110.2	157.8±80	292.2±124.4	125 [0 ; 250]
	M	250[125 ; 250]	156[110 ; 220]	300±140.5	125 [125 ; 250]
	Total^++^	242±120.2	156±96.3	300±133.4	125 [62.5 ; 250]
7 à 9 ans	F	170.8±79.6	110[55 ; 220]	300±180.9	100 [0 ; 250]
	M	125 [125 ; 250]	110±37.7	252.6±97.1	111.1±75.1
	Total^+++^	172.1±65.4	117.9±66.5	269.1±130.9	125[0 ; 125]
10 à 16 ans	F	178.8±77.5	130.6±44.3	373.1±168.1	125[93.8 ; 187.5]
	M	125 [125 ; 250]	128.3±62.1	240[240 ; 360]	112.5±71
	Total^++++^	176.1±91.6	129.3±55.1	240[240 ; 480]	125±91.3

M : masculin, F : féminin, g : gramme

+ p<= 0.001

++ p= 0.016

+++ p= 0.026

++++ p= 0.44

**Tableau 4:** Facteurs associés à la consommation d’au moins 3 produits laitiers par jour en analyse univariée

	Analyse univariée				
	1 à 2 produits laitiers	≥ 3 produits laitiers	OR cru	IC 95%	p
Age maternel	36±8.8	35.5±7.7	0,9	0.9 ; 1	0,52
Données manquantes	5	5			
**Niveau d’instruction**					
Analphabète	66 (55.5)	36 (32.4)	0.1	0.03 ; 0.3	<0.001
Primaire	36 (30.3)	27 (24.3)	0.1	0.04 ; 0.5	0.002
Secondaire	13 (10.9)	29 (26.1)	0.4	0.1 ; 1.6	0.24
Universitaire	4(3.4)	19 (17.1)	1		
Données manquantes	7	10			
**Profession de la mère**					
Non	115 (95)	103 (89.6)	0,4	0.1 ; 1.2	0,1
Oui	6 (5)	12 (10.4)	1		
Données manquantes	5	6			
**Niveau socioéconomique familial**					
Bas	97 (80.2)	64 (56.1)	1		
Moyen	23 (19)	46 (40.4)	3	1.6 ; 5.4	<0.001
Haut	1 (0.8)	4 (3.5)	6.1	0.6 ; 55.4	0.1
Données manquantes	5	7			
Age de l’enfant	7.5±4.2	6.2±4	0.9	0.8 ; 0.9	0.01
Données manquantes	0	1			
**IMC**					
< 3°percentile	38 (31.7)	15 (14.7)	1		
normal	72 (60)	65 (63.7)	2.5	1.2 ; 5	0.01
>90° percentile	10 (8.3)	22 (21.6)	6	2.3 ; 15.7	<0.001
Données manquantes	6	16			
**Sexe**			0.8	0.5 ; 1.3	0.5
F	61 (48.8)	53 (44.5)			
M	64 (51.2)	66 (55.5)			
Données manquantes	1	2			
Poids	25.6±14.7	21.5±11.9	1	0.9 ; 1	0.01
Données manquantes	2	5			
Taille	116.6±32	110.5±33.4	0.99	0.98 ; 1	0.2
Données manquantes	6	10			
**Activité physique: fréquence/semaine**					
0	103 (89.6)	98 (89.1)	0.8	0.2 ; 2.7	0.7
1 à 2	4 (3.5)	4 (3.6)	0.8	0.1 ; 5.1	0.8
2 à 3	3 (2.6)	2 (1.8)	0.5	0.06 ; 4.7	0.6
> 3	5 (4.3)	6 (5.5)	1		
Données manquantes	11	11			

OR : odds ratio, IC : intervalle de confiance, F : féminin, M : masculin

**Tableau 5:** Facteurs indépendamment associés à la consommation d’au moins 3 produits laitiers par jour

	Analyse multivariée		
	OR ajusté	IC 95%	p
**Niveau d’instruction maternel**			
Analphabète	0.1	0.02 ; 0.6	0.008
Primaire	0.1	0.02 ; 0.6	0.009
Secondaire	0.3	0.06 ; 2	0.2
Universitaire	1		
**Niveau socioéconomique familial**			
Bas	1		
Moyen	1.7	0. 8 ; 3.7	0.2
Haut	2.1	0. 2 ; 26	0.6
Age de l’enfant	0.9	0. 8 ; 1	0.1
**IMC**			
< 3°percentile	1		
normal	2.1	0.9 ; 4.8	0.08
>90° percentile	3.9	1.3 ; 11.9	0.01

OR : odds ratio, IC : intervalle de confiance

## Discussion

Notre étude a certaine limite, notre échantillon n’est pas représentatif de la population marocaine, en outre il y a un risque de biais mémoire du questionnaire fréquentiel. Cette enquête alimentaire sur la consommation des produits laitiers est la première réalisée dans notre établissement à notre connaissance chez l’enfant âgé de 2 à 16 ans. Les trois quarts des enfants consommaient les produits laitiers chaque jour avec comme chef de file le lait et les yaourts. La consommation du fromage et des autres produits laitiers était moins fréquente. Le lait était principalement consommé au moment du petit déjeuner mais sa consommation était inférieure à 500 ml par jour chez la majorité de nos enfants, ce qui était rapporté par d’autres études [10–14]. Une cohorte avait démontré une consommation quotidienne du lait chez l’enfant de 1 à 3 ans de 200 g par jour, chez l’enfant de 4 à 8 ans de 200 g par jour, chez les garçons de 9 à 13 ans de 221 g par jour et chez les filles de 9 à 13 ans de 163 g par jour [2, 15]. La consommation du lait chez l’enfant âgé de 1.5 à 4.5 ans était de 303 ml/ jour sous forme de lait entier et de 186 ml/ jour réduit en matières grasses, chez l’enfant âgé de 4 à 18 ans était de 171 ml/ jour du lait entier et de 149 ml/ jour du lait réduit en matières grasses [12]. La diminution de la consommation du petit déjeuner pourrait expliquer la baisse de la consommation du lait qui était principalement consommée lors du petit déjeuner.

En France, la consommation du petit déjeuner avait diminué avec l’âge de 87%, 71%, 50% respectivement chez les enfants âgés de 3-10 ans, 11-14 ans et 15-17 ans [16]. La consommation d’au moins 3 produits laitiers par jour était insuffisante et inférieure aux recommandations chez les enfants des différentes tranches d’âge même s’il était relativement plus élevé chez les enfants jeunes de moins de 6 ans. Les enfants consommaient au moins 3 produits laitiers par jour dans 57.8% chez les enfants âgés de 2 à 3 ans, 53.6% chez les enfants âgés de 4 à 6 ans, 40% chez les enfants âgés de 7 à 9 ans et 41.2% chez les enfants âgés de 10 à 16 ans. En France, deux études nationales sur les consommations alimentaires réalisées en 1999 et 2007 ont démontré un déclin de la consommation des produits laitiers en particulier le lait chez l’enfant et l’adolescent âgés de 3 à 14 ans de 11% [7, 16]. L’apport total du lait, du fromage et des produits laitiers frais avait baissé de 10%, 12% et 9.1% respectivement chez l’enfant âgés de 3 à 10 ans, 11 à 14 ans et 15 à 17 ans selon la même étude. En Allemagne, l’étude nutritionnelle longitudinale réalisée de 1986 à 2001 chez l’enfant de 1 à 13 ans, avait démontré une baisse de la consommation du lait chez l’enfant de 1 à 3 ans de - 6.5g/j/année d’étude, chez l’enfant de 4 à 13 ans de -2.8 à -7.4 g/j/année d’étude avec une augmentation de la consommation du fromage de + 0.2 à 0.7 g/j/année d’étude et du yaourt de +2.4 à 3.3 g/j/année d’étude [2].

La consommation de moins de 3 produits laitiers par jour était corrélée de façon négative au faible niveau socio-économique familial dans notre étude en analyse univariée. Le faible niveau socioéconomique avait un impact négatif sur l’apport calcique chez les enfants d’âge préscolaire [17]. Le faible niveau d’éducation maternel était un facteur de risque de la consommation de moins de 3 produits laitiers par jour après ajustement statistique dans notre étude. Le haut niveau d’éducation des parents était associé positivement à la consommation du lait et des produits laitiers [18]. La consommation du lait diminue avec l’âge de l’enfant dans notre étude en analyse univariée, ce qui a été rapporté par plusieurs études dans la littérature [14, 19]. Les enfants de sexe masculin consommaient plus de produits laitiers par jour que les filles mais cette différence n’est pas statistiquement significative. Plusieurs études dans la littérature [20, 21], avait rapporté l’augmentation de la consommation des produits laitiers des garçons et l’expliquaient en partie par les besoins physiologiques augmentés. L’indice de masse corporelle normal et supérieur au 90° percentile était un facteur indépendamment associé à la consommation de 3 produits laitiers minimum par jour dans notre enquête. L’association entre la consommation des produits laitiers et l’indice de masse corporelle était controverse dans la littérature. La consommation du lait ou des produits laitiers aux USA chez l’enfant de 2 à 10 ans était associée à un percentile d’indice de masse corporelle élevé [22]. L’obésité n’était pas associée positivement à la consommation du lait et des produits laitiers entier ou réduit en matière grasse chez l’enfant [23, 24] et l’adolescent dans d’autres études [25]. Les auteurs expliquaient que l’association de l’obésité et la consommation des produits laitiers était plutôt lié à des facteurs sociaux notamment le faible niveau socioéconomique qui était classiquement décrit dans la littérature comme un facteur de risque de l’obésité [26, 27]. L’activité physique avec une alimentation variée y compris la consommation des produits laitiers était associée négativement au surpoids à chez l’enfant [28]. Dans notre étude, cette association n’était pas constatée, probablement parce que 89.9% des enfants n’avaient pas d’activité physique régulière

## Conclusion

La consommation du lait et des autres produits laitiers était inappropriée dans toutes les tranches d’âge mais très inférieure aux recommandations en particulier chez l’enfant âgé de 7 à 9 ans et l’adolescent de 10 à 16 ans. Le faible niveau d’éducation maternel avait un impact négatif sur la consommation des produits laitiers. L’indice de masse corporelle supérieur au 90° percentile était associé positivement à une consommation des produits laitiers. Un programme de sensibilisation régional voire national y compris scolaire doit être instauré tout en ayant deux principaux axes ; informer les parents et les enfants sur l’importance de la consommation des produits laitiers comme la principale source alimentaire du calcium pour une bonne croissance staturale particulièrement en péripubertaire et promouvoir une activité physique régulière. Cette action devrait cibler en premier les enfants issus de famille de faible niveau socioéconomique ou de mères ayant un faible niveau d’éducation.

### Etat des connaissances actuelles sur le sujet

- Les enquêtes de consommation dans de nombreux pays révèlent une faible consommation du lait et de ses dérivés qui n’atteint pas les apports nutritionnels recommandés chez l’enfant avec une tendance à la diminution de cette consommation avec l’âge;
- Une association positive existe entre le pic de la densité minérale osseuse à l'âge adulte et la consommation de produits laitiers pendant l’enfance.

### Contribution de notre étude à la connaissance

- Notre étude montre que la consommation du lait et des autres produits laitiers est inappropriée dans toutes les tranches d’âge mais très inférieure aux recommandations en particulier chez l’enfant âgé de 7 à 9 ans et l’adolescent de 10 à 16 ans;
- Le faible niveau socioéconomique et le faible niveau d’éducation maternel ont un impact négatif sur la consommation des produits laitiers;
- Il est donc important d’établir un programme de sensibilisation régional voire national y compris scolaire ciblant les parents et les enfants particulièrement ceux issus de famille de faible niveau socioéconomique ou de mères ayant un faible niveau d’éducation, sur l’importance des produits laitiers comme principale source alimentaire de calcium pour une bonne croissance staturale en particulier en péripubertaire avec une promotion de l’activité physique régulière.

## Conflits d’intérêts

Les auteurs ne déclarent aucun conflit d'intérêts.
